# Serum RelB is correlated with renal fibrosis and predicts chronic kidney disease progression

**DOI:** 10.1002/ctm2.362

**Published:** 2021-05-21

**Authors:** Donglin Sun, Ningxia Xie, Xi Wang, Wenquan Wu, Xiu‐Yong Li, Xiangqiu Chen, Guojun Qian, Cuifeng Li, Haohao Zhang, Yuhang Jiang, Deji Ye, Dandan Liu, Yiming Hu, Jingyao Wang, Weifeng Chen, Qiumei Zhao, Min Zeng, Junwei Zhang, Li Wang, Xiaoren Zhang

**Affiliations:** ^1^ Affiliated Cancer Hospital and Institute of Guangzhou Medical University Guangzhou Municipal and Guangdong Provincial Key Laboratory of Protein Modification and Degradation State Key Laboratory of Respiratory Disease; ^2^ Shenzhen Longhua District Central Hospital Nephrology Department; ^3^ Southern Medical University Affiliated Longhua People's Hospital Clinical Laboratory; ^4^ NO.2 People's Hospital of Fuyang City Fuyang 236015 China; ^5^ Shenzhen Hospital Southern Medical University Urology Department; ^6^ Shanghai Institute of Nutrition and Health Shanghai Institutes for Biological Sciences University of Chinese Academy of Sciences Chinese Academy of Sciences CAS Key Laboratory of Tissue Microenvironment and Tumor; ^7^ Nephrology Department Southern Medical University Affiliated Longhua People's Hospital

Dear Editor,

Chronic kidney disease (CKD) with characteristics of progressive deterioration of renal function is regarded as one major public health problem around the world. The patients are often diagnosed at advanced stages with most kidney functions lost, since they have few signs or symptoms at early stage. Renal fibrosis is the pathologic hallmark and prognostic indicator for CKD. The effective methods except biopsy for early detection of renal fibrosis are required to improve the diagnosis of CKD. Our data revealed that RelB can not only discriminate CKD patients from normal subjects but also can distinguish CKD patients at different stages. We prove that RelB is capable to work as a promising serum biomarker to diagnose and monitor the renal fibrosis in CKD patients.

Renal inflammation is actively participating in the evolution of renal fibrosis and CKD.^1^ In kidney tubular cells, hyperactivation of noncanonical NF‐κB signaling induced by TWEAK, TNF‐like weak inducer of apoptosis, contributes to inflammatory responses.^2^ Through analyzing a whole transcriptome RNA sequencing of mouse renal fibrosis model with unilateral ureteral obstruction (UUO), we found that RelB, the key transactivator of noncanonical NF‐κB signaling pathway, was profoundly elevated in fibrotic renal tissues (Figure S1).^3^ Therefore, we established UUO model to validate the upregulation of RelB in the progressive renal fibrosis. The results showed a consistent and significant increase in RelB along with α‐SMA and Col1α1 at mRNA and protein levels in fibrotic kidneys after UUO (Figure 1A and B), and the level of *RelB* mRNA was positively correlated with *α‐SMA*, *Col1α1*, and *Tgfβ1* (Figure S2A–C), while the RelB protein was correlated with fibrosis (Figure S2D and E). Then, we measured the concentration of serum RelB in mice with ureteric ligation and found it increased significantly from the 8th day after UUO as well (Figure 1C). The immunohistochemical analysis further showed that RelB, specifically expressed in the renal tubular epithelial cells, gradually increased with the fibrosis progression (Figure 1D‐F ), and was positively correlated with the severity of fibrosis reflected by the Masson's staining (*r* = 0.5861, *p* = 0.0106) (Figure 1G ). Correspondingly, the concentration of serum RelB protein was positively correlated with the severity of fibrosis (*r* = 0.5726, *p* = 0.0130) (Figure 1H) and the renal RelB staining (*r* = 0.8260, *p* < 0.0001) (Figure S2F). These data clearly manifested that RelB is gradually induced in fibrotic kidney after UUO in mice and the expression of RelB can be an indicator of renal fibrosis.

**FIGURE 1:**
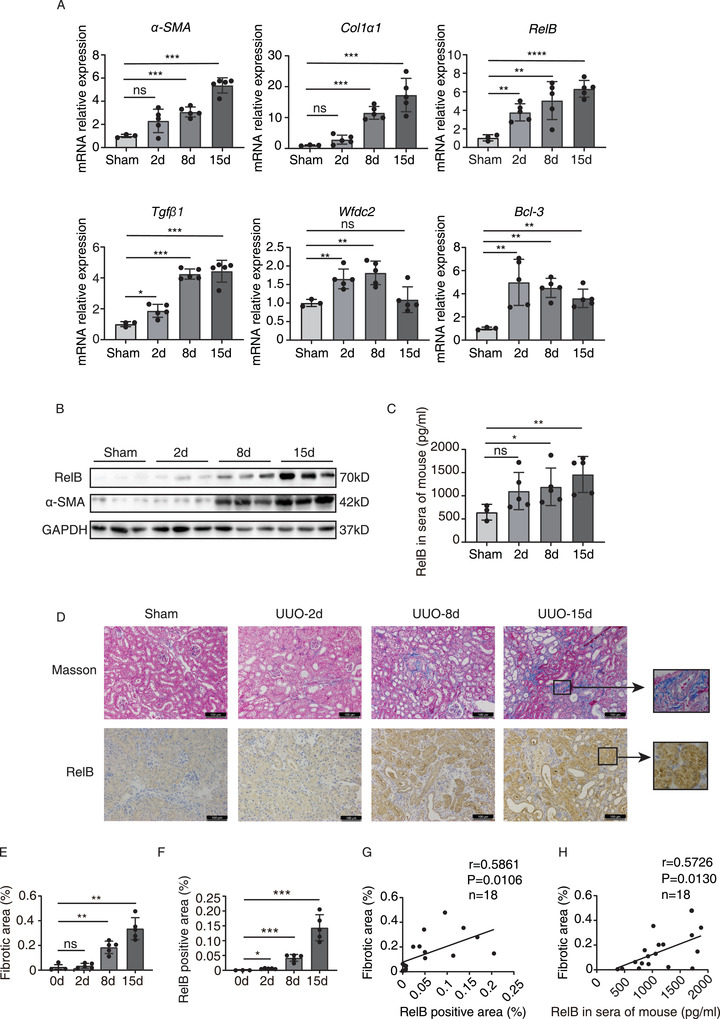
RelB is induced after UUO and correlated with kidney fibrosis in mice. (A) qRT‐PCR analysis of *RelB*, *Wfdc2*, *α‐SMA*, *Col1α1*, *Bcl‐3*, and *Tgfβ1* in obstructive nephropathy at indicated time points after UUO. Sham group includes three mice while each UUO group includes five mice. (B) Western blot analysis of renal RelB and α‐SMA in obstructive nephropathy at time points of 0 (sham group), 2, 8 and 15 days after UUO. (C) ELISA detection of serum RelB in obstructive nephropathy after UUO at indicated time points. (D) Representative images show renal collagen deposition by Masson's trichrome staining (blue) and RelB expression by immunohistochemical staining at different time points of UUO. Scale bars: 100 μm. (E) Quantitative analysis of the positive areas of Masson's trichrome staining in indicated groups. (F) Quantitative analysis of the positive staining for RelB in indicated groups. (G) Scatter plot with linear regression shows a correlation between tissue RelB expression and kidney fibrosis. *r* = 0.5861; *p* = 0.0106; *n* = 18. (H) Correlation between RelB serum content and kidney fibrosis was implicated by the scatter plot with linear regression. *r* = 0.5726; *p* = 0.0130; *n* = 18. Data were exhibited as mean ± SD. *p < 0.05, **p < 0.01, ***p < 0.001, ****p < 0.0001

A cohort of the biopsy‐proven CKD patients was subsequently studied. Kidney biopsy specimens were collected from 34 CKD patients for immunohistochemical staining (Figure 2A). Among these samples, the protein expressions of RelB were disclosed with positive correlations with the intensity of kidney fibrosis (*r* = 0.6994, *p* < 0.0001) (Figure 2B). We collected serum from 15 patients among these 34 patients and measured the content of RelB in sera. The serum RelB level was positively correlated with the intensity of renal fibrosis as well (*r* = 0.8388, *p* = 0.003) (Figure 2C). We next analyzed more blood samples including 32 CKD patients and 60 healthy people, and found that the concentration of RelB and HE4 (a literature reported serum biomarker for renal fibrosis in CKD^4^, ^5^, ^6^, ^7^) in sera from CKD patients expressed higher levels than from healthy controls (Figure 2D, Table 1). More importantly, we found the serum RelB or HE4 level was also significantly correlated with the renal function. They were positively correlated with serum creatinine (SCr) and blood urea nitrogen (BUN), and negatively correlated with estimated glomerular filtration rate (eGFR) (Figure 2E and F, Figure S3), indicating that it can reflect the injury of renal function. In addition, the serum RelB level was positively correlated with the serum HE4 level (Figure 2G). Being evaluated within the receiver operating curve (ROC) analysis afterwards, serum RelB and HE4 showed predictive significance for CKD detection. The area under the curve (AUC) value of RelB and HE4 was 0.873 and 0.849, respectively, while the AUC of RelB‐HE4 combination was 0.920 (Figure 2H). In addition, the Hosmer–Lemeshow test was used to check the calibration of predictive model (Figure S4A). Summarily, it suggests that RelB is likely to be a better predictor of renal fibrosis and CKD compared with HE4, while its predictive accuracy can be improved when combined with HE4 (Table S1).

**FIGURE 2:**
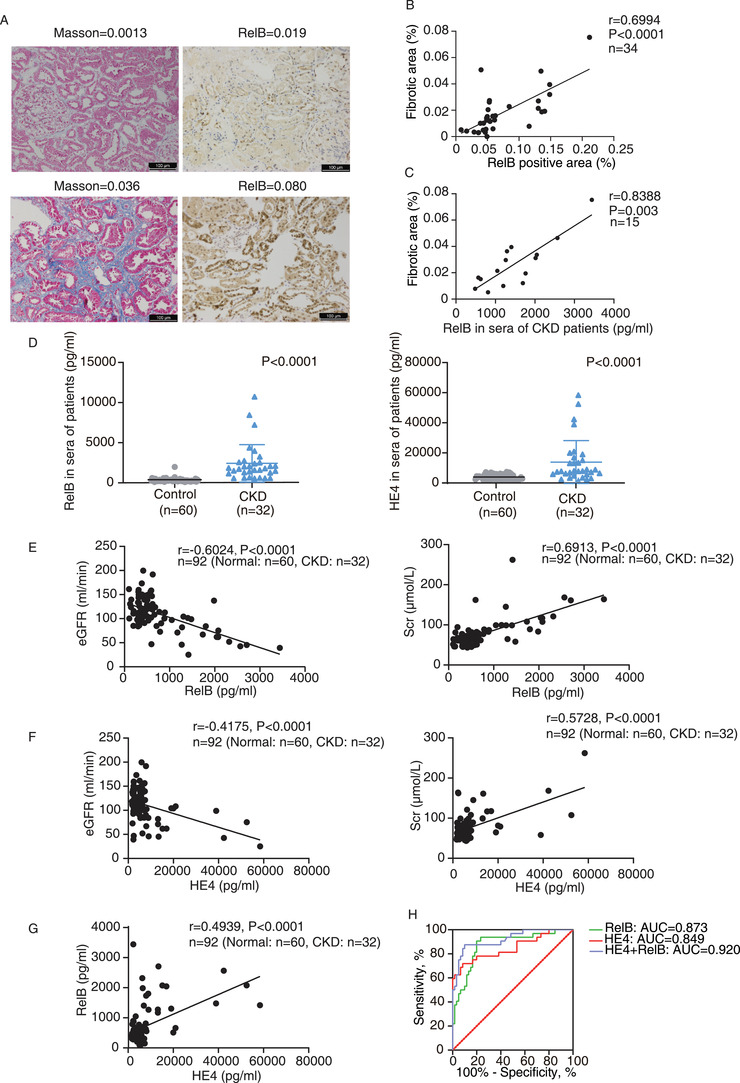
The expression of RelB is correlated with kidney fibrosis and renal function indexes in CKD patients and can discriminate CKD patients from the healthy control. (A) The representative images show renal collagen deposition by Masson's trichrome staining (blue) and RelB expression by immunohistochemical staining in kidney biopsies of CKD patients. The mean densities of Masson and RelB in kidney tissues were given at the top of each image. Scale bar = 100 μm. (B) Scatter plot with linear regression shows a correlation between tissue RelB expression and kidney fibrosis. *r* = 0.6994; *p* < 0.0001; *n* = 34. (C) The content of RelB in CKD patients was correlated with kidney fibrosis through the scatter plot with linear regression. *r* = 0.8388; *p* = 0.003; *n* = 15. (D) The serum RelB and HE4 levels in the patients (CKD, *n* = 32) and the healthy subjects (control, *n* = 60). (E and F) Scatter plot with linear regression shows that serum RelB (E) and HE4 (F) were respectively correlated with eGFR and SCr among CKD patients and healthy group. (G) Correlation between RelB and HE4 in serum of CKD patients and healthy people. The Spearman correlation coefficient (*r*) and *p* value are shown. (H) The ROC analysis of potential biomarkers including RelB, HE4, and their combination evaluates their ability to discriminate CKD patients from the healthy control. The associated AUC values are indicated

**TABLE 1:** Characteristics of participants

	Normal control (*n* = 60)	CKD I and II (*n* = 25)	CKD III and IV (*n* = 7)
Age, years	30.10 ± 9.00	40.12 ± 11.71^***^	33.86 ± 10.24
Gender, M/F	30/30	20/5	5/2
Weight, kg	60.37 ± 9.47	68.67 ± 8.74^**^	66.60 ± 12.78
Scr, μmol/L	62.82 ± 12.32	81.35 ± 20.15^***^	169.10 ± 44.04^***,##^
BUN, mmol/L	4.06 ± 0.88	5.75 ± 2.43^***^	8.76 ± 4.07^***,#^
eGFR, mL/min	122.72 ± 25.01	103.79 ± 29.94^*^	42.49 ± 8.63^***,##^
Relb, pg/mL	408.67 ± 256.37	1017.25 ± 498.95^***^	2066.176 ± 972.93^***,##^
HE4, pg/mL	3946.23 ± 1725.18	12,302.56 ± 11,578.87^***^	22,147.6 ± 19,185.18^***^

*Note*: Compared with control, * *p* < 0.01, ** *p* < 0.001, and *** *p* < 0.0001, Student's *t*‐test and c2 test were used. Compared with CKD I and II, #*p* < 0.05, #### *p* < 0.0001, Student's *t*‐test and c2 test were used.

Abbreviations: BUN, blood urea nitrogen; CKD, chronic kidney disease; eGFR, estimated glomerular filtration rate; Scr, serum creatinine.

When dividing the 32 CKD patients into two groups: CKD I & II and CKD III & IV, we showed that not only the level of serum RelB was significantly upregulated in the early‐stage patients, but it also increased remarkedly along with the progression of stages (Figure 3A). Nevertheless, no significant differences were identified in the levels of serum HE4 between stage I and II and stage III and IV of CKD patients (Figure 3A). The serum levels of RelB and HE4 were observed with significant correlations (Figure 3D) and were additionally associated with SCr, BUN, or eGFR (Figure 3B and C, Figure S3). Then, the ROC analysis indicated the ability of RelB, but not HE4, to discriminate the CKD I & II patients with the CKD III & IV ones (Figure 3E). The Hosmer–Lemeshow test further indicated RelB or RelB‐HE4 combination was better calibrated in distinguishing CKD I & II and CKD III & IV patients (Figure S4B). These results indicated that the serum RelB can well discriminate CKD patients at different stages.

**FIGURE 3:**
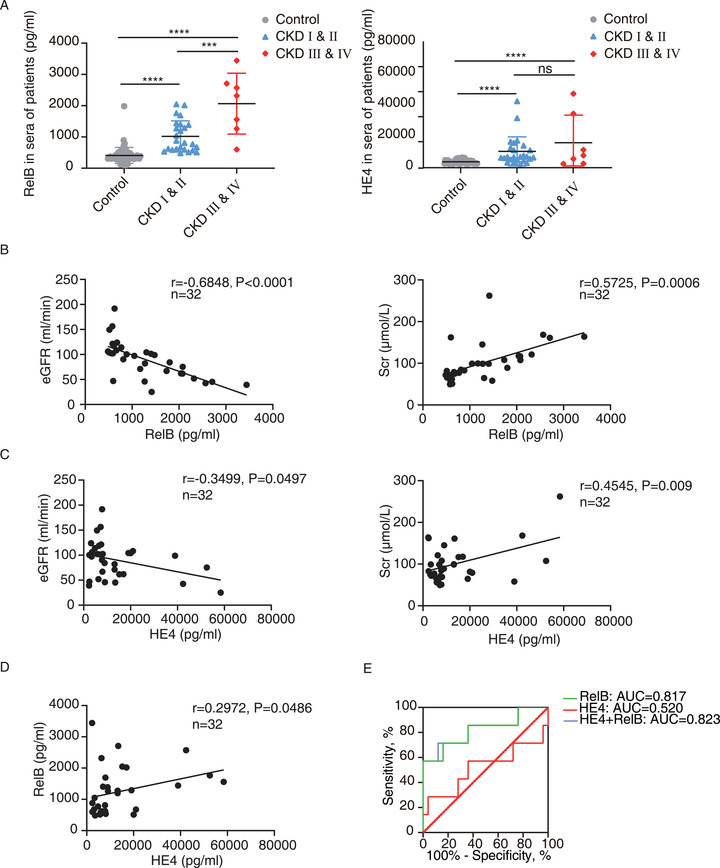
The serum RelB can discriminate CKD patients at different stages. (A) The serum RelB and HE4 levels in the CKD patients with different stages (CKD I and II, *n* = 25; CKD III and IV, *n* = 7) and the normal controls (control, *n* = 60). (B–D) Scatter plot with linear regression implied the content of serum RelB (B), HE4 (C), as well as the combination of RelB and HE4 serum levels (D) had correlations with eGFR and SCr in CKD patients. The Spearman correlation coefficient (*r*) and *p* value are shown. (E) The ROC analysis of potential biomarkers including RelB, HE4, and their combination evaluates their ability to distinguish CKD I and II patients from CKD III and IV ones. The associated AUC values are indicated. Data were exhibited as mean ± SD. ***p < 0.001, ****p < 0.0001

Recently, accumulated evidence has revealed the importance of noncanonical NF‐κB signaling in regulating both innate and adaptive immune responses, as well as the pathogenesis of inflammatory diseases.^8^, ^9^, ^10^ The present study revealed that its pivotal transcription factor RelB has the potential as the biomarker for renal fibrosis identification at CKD early stages. Since it can distinguish CKD patients from healthy controls and discriminate CKD patients at different stages, it might be useful to supplement the existing biochemical indexes of kidney disease. Due to the sampling limitation in this study, it is ideal to expand the patient samples for comparative study on CKD patients with different kidney disease types and for better evaluation of the diagnostic value of RelB in CKD.

## CONFLICT OF INTEREST

The authors declare no conflict of interest.

## AUTHOR CONTRIBUTIONS

Xiaoren Zhang, Ningxia Xie, and Li Wang designed the research. Donglin Sun and Ningxia Xie performed the experiments and interpreted the data. Donglin Sun, Ningxia Xie, Xi Wang, Wenquan Wu, Xiuyong Li, and Jingyao Wang collected and prepared human samples. Xiaoren Zhang, Li Wang, Donglin Sun, Ningxia Xie, Yiming Hu, Xiangqiu Chen, Guojun Qian, Cuifeng Li, Haohao Zhang, Yuhang Jiang, Dandan Liu, Weifeng Chen, Qiumei Zhao, and Deji Ye wrote and/or reviewed the manuscript. Li Wang, Min Zeng, and Junwei Zhang applied ethic approval and signed informed consent. All authors approved the final manuscript.

## FUNDING INFORMATION

This work was supported by the National Key R&D Program of China (2018YFA0107500), the National Natural Science Foundation of China (91949102, 91742113), and the Guangzhou Key Medical Discipline Construction Project Fund.

## Supporting information

Supporting Information

**Supplemental Figure 1. Altered expression of genes in NF‐κB and TGF‐β signaling pathways between UUO groups and sham group**. The heatmap shows the renal tissue transcriptome‐wide changes in gene expression among three different groups (sham‐operated group, 2 days post‐UUO group and 8 days post‐UUO group). Normalized read counts values are recorded. 34 genes related to NF‐κB and TGF‐β signaling pathways were analyzed from a set of RNA‐seq data available at the NCBI GEO repository. GEO accession: GSE79443.
**Supplemental Figure 2. The correlation between RelB and kidney fibrosis at mRNA levels and protein levels. (A, B, C)** Scatter plot with linear regression shows a correlation between mRNA expression of RelB and kidney fibrosis in obstructive nephropathy after UUO. **(D)** Quantification of the RelB Western blot results. **(E)** Correlation between RelB protein levels measured by Western blot and kidney fibrosis. *r* = 0.8854; *p* = 0.0001; *n* = 12. **(F)** Correlation between renal RelB staining and serum RelB, *r* = 0.8260; *p* < 0.0001; *n* = 18. Data were exhibited as means ± S.D. *p < 0.05 and **p < 0.001.
**Supplemental Figure 3. The correlation between the serum RelB and BUN. (A)** Scatter plot with linear regression shows the serum RelB and HE4 levels are positively correlated with BUN in healthy controls and CKD patients. **(B)** Scatter plot with linear regression shows the serum RelB levels are positively correlated with BUN in CKD patients. However, correlation analysis revealed no significant associations between the serum HE4 and BUN. The Spearman correlation coefficient (*r*) and *p* value are shown.
**Supplemental Figure 4. Calibration of the model. (A)** The calibration curve and Hosmer‐Lemeshow test for the predictive ability of RelB (*χ* ^2 ^= 13.16, *p* = 0.106), HE4 (*χ* ^2 ^= 14.12, *p* = 0.079) and the RelB + HE4 combination (*χ* ^2 ^= 9.11, *p* = 0.333) in healthy group and CKD patients demonstrated good agreement with observation. **(B)** Similarly, the predictive ability of the RelB (*χ* ^2 ^= 10.61, *p* = 0.225), HE4 (*χ* ^2 ^= 10.31, *p* = 0.244) and the RelB + HE4 combination (*χ* ^2 ^= 10.35, *p* = 0.241) between early‐stage CKD patients (I & II) and advanced‐stage CKD patients (III & IV) showed good agreement with observation by the calibration curve and Hosmer‐Lemeshow test.

Table S1
