# Supplementary material for: Serum RelB is correlated with renal fibrosis and predicts chronic kidney disease progression
Source: Clin Transl Med. 2021 May 21;11(5):e362. doi: 10.1002/ctm2.362 (PMC8140188; doi:10.1002/ctm2.362)
Supplement: Supplementary file 1 — Supporting Information [file CTM2-11-e362-s003.docx]

**Supporting Information**

**Methods**

**Human blood and kidney biopsies samples**

Human blood and kidney specimens were clinically redundant samples obtained from diagnostic renal biopsies performed at the Southern Medical University Affiliated Longhua People’s Hospital from 2019 to 2020, including 32 blood samples and 34 renal puncture samples of CKD patients, and 60 control blood samples of healthy volunteers. The blood samples were firstly collected, and then centrifuged to remove the blood cells after standing for a while. The serum samples were immediately processed and stored at -80°C. The paraffin-embedded human kidney biopsy sections (5 μm thickness) were prepared using a routine procedure. The patients who had received corticosteroids or other immunosuppressive therapies for the kidney disease, or had systemic diseases were excluded from the study.

**Mouse model of UUO**

Male C57BL/6 mice (8 weeks old) used to establish the unilateral ureteral obstruction (UUO) renal fibrosis model were purchased from the Shanghai Laboratory Animal Center, Chinese Academy of Sciences. Prior to surgery, mice were anesthetized with 1.25% 2,2,2-Tribromoethanol (Sigma-Aldrich, T48402). Then, complete UUO was conducted by double-ligating the left ureter by 4-0 silk following the abdominal incision. The ureters of sham operated mice were exposed, but not ligated. After UUO, the experimental mice were sacrificed on day 2, 8, and 15, respectively, the serum and the kidney tissues were collected for further analysis. The mice were housed and bred at specific-pathogen-free (SPF) facility. All animal experiments were conducted in accordance with NIH Guide for the Care and Use of Laboratory Animals (National Academies Press, 2011), and animal protocols were approved by the Institutional Biomedical Research Ethics Committee of the Shanghai Institutes for Biological Sciences at the Chinese Academy of Sciences.

**RNA extraction and real-time RT-PCR**

The kidney tissue was homogenized with TRIzol Reagent (Life technologies, 15596-026) and the total RNA was extracted according to the manufacturer’s instructions. Then the RNA was converted to cDNA using PrimeScript™ RT reagent Kit with gDNA Eraser (TaKaRa, RR047A) according to the manufacturer’s instructions. Real time RT-PCR was conducted with SYBR® Premix Ex Taq ™ (TaKaRa, RR420A) on the Q7 RT-PCR detection system (Life technologies). The 2^−ΔΔCT^ cycle threshold method was used to calculate the relative change in expression. Results were normalized to the expression of *β-actin* mRNA. The primers used are listed below:

| **Primer name** | **Sequence (5’-3’)** |
| --- | --- |
| mRelB-F | ACTGGATGCCCAGGTTGTTA |
| mRelB-R | CCTGGTGTGGAAGGACTGG |
| mBcl-3-F | cactttccgctgctgaacct |
| mBcl-3-R | CTGAGTATTCGGTAGACAGC |
| mWfdc-2-F | AACCAATTACGGACTGTGTGTT |
| mWfdc-2-R | TCGCTCGGTCCATTAGGCT |
| mACTA2-F | GTTCAGTGGTGCCTCTGTCA |
| mACTA2-R | ACTGGGACGACATGGAAAAG |
| mCol1α1-F | GCTCCTCTTAGGGGCCACT |
| mCol1α1-R | CCACGTCTCACCATTGGGG |
| mTgfb1-F | CACGTGGAAATCAACGGGAT |
| mTgfb1-R | GCGCACAATCATGTTGGACA |
| mActb-F | ATCTACGAGGGCTATGCTCTCC |
| mActb-R | CTTTGATGTCACGCACGATTTCC |

**RelB and HE4 ELISA analysis**

The serum human RelB (FineTest, EH1905), the serum mouse RelB (Abbexa, abx353124) and the serum human HE4 (R&D, DHE400) levels were determined by ELISA kits according to the manufacturers’ instructions. Each sample was measured in duplicate.

**Western blot analysis**

Kidney tissues were washed with ice-cold PBS, homogenized and lysed in RIPA lysis buffer (Beyotime, P0013F) supplemented with protease inhibitors cocktail (Roche Diagnostics, 4693116001) and PMSF (Beyotime, ST506). The lysates were incubated on ice for 30 min before centrifuging at 13 000 g for 15 min, and then the supernatants were collected and stored at -80 °C. The concentration of total protein was estimated using BCA protein assay kit (Thermo Fisher Scientific, 23225). The protein samples (10 - 20 μg) diluted in 5 × loading buffer and boiled for 5 minutes were separated by 12% SDS-polyacrylamide gel electrophoresis (SDS-PAGE) and transferred onto PVDF membranes (Millipore) for following probing with specific antibodies. The immune-reactive proteins were visualized by enhanced chemiluminescence detection system (Millipore). The primary antibodies used were as follows: Anti-RelB (rabbit; Abcam, ab180127), Anti-alpha smooth muscle actin (anti-α-SMA; rabbit; Abcam, ab5694), Anti-GAPDH (KANGCHEN, KC-5G4).

**Histology and immunohistochemistry**

The kidneys were fixed in 4% paraformaldehyde fix solution (Beyotime, P0099-500ml) and embedded in paraffin. Paraffin-embedded mouse kidney sections (3 μm thickness) were prepared by a routine procedure. The sections were stained with hematoxylin and eosin (H&E) and Masson trichrome reagent (Servicebio, G1006) separately. For the immunohistochemical staining, the sections were deparaffinized and rehydrated, followed with antigen retrieval and blocking. Then the tissue sections were incubated with diluted anti-RelB primary antibodies (Abcam, ab180127) at 4 °C overnight. The secondary antibodies were applied, and diaminobenzidine (DAB) solution was used as a chromogen. Finally, the sections were counterstained with hematoxylin to identify nuclei. The images were photographed by a general optical microscope (Carl Zeiss) and analyzed using Image-Pro Plus 6.0 software (Media Cybernetics Inc).

**Statistically analysis**

The animal experiments were performed more than 2 independent times. Results in figures are pooled from more than 3 mice or represent independent experiment with biological replicates. Data are presented as the mean ± SD, unless stated otherwise. The statistical significance was calculated by two-tailed Student’s t test for the differential RNA and protein expression analyses. The correlations of RelB with renal fibrosis or renal function indexes were calculated by linear regression analyses. The diagnostic values of RelB were evaluated by the receiver-operating characteristic (ROC) curve analyses and the area under the ROC curve (AUC-ROC). The optimal cut-off point was determined by Youden’s index. Statistical analysis was performed by using GraphPad Prism 5 (GraphPad Software, La Jolla, CA, USA). P value less than 0.05 were considered statistically significant.

**Ethic approval and consent to participate**

Our research did not involve and disclose any privacy information of patients, and did not concern any diagnosis or treatment for the subjects. All patients were recruited after institutional review board approval and signed informed consent. The study was approved by the Ethics Committee of the Southern Medical University Affiliated Longhua People’s Hospital. There would not be any risk or effect on the participates.
