# Supplementary figures and images for: Serum RelB is correlated with renal fibrosis and predicts chronic kidney disease progression
Source: Clin Transl Med. 2021 May 21;11(5):e362. doi: 10.1002/ctm2.362 (PMC8140188; doi:10.1002/ctm2.362)

Supplemental Figure 1

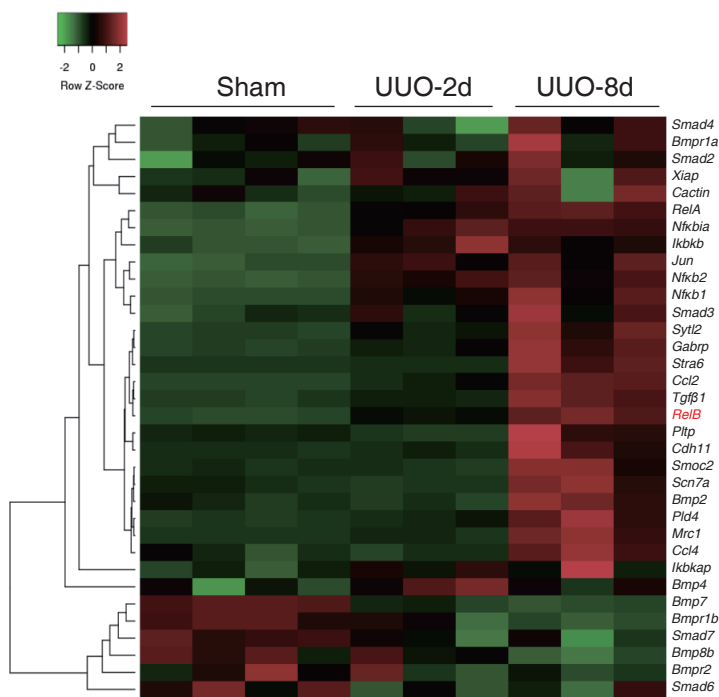

Supplemental Figure 2

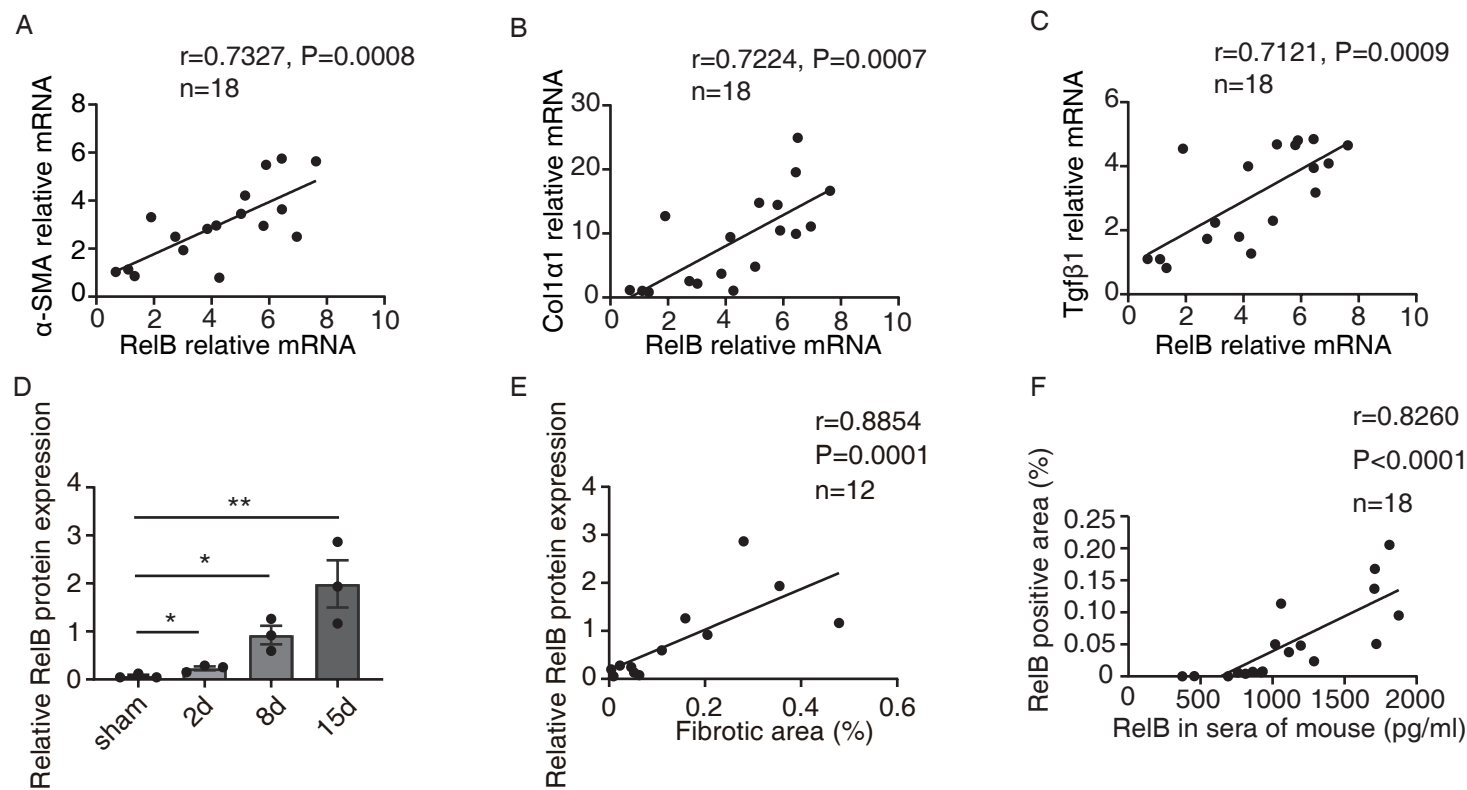

Supplemental Figure 3

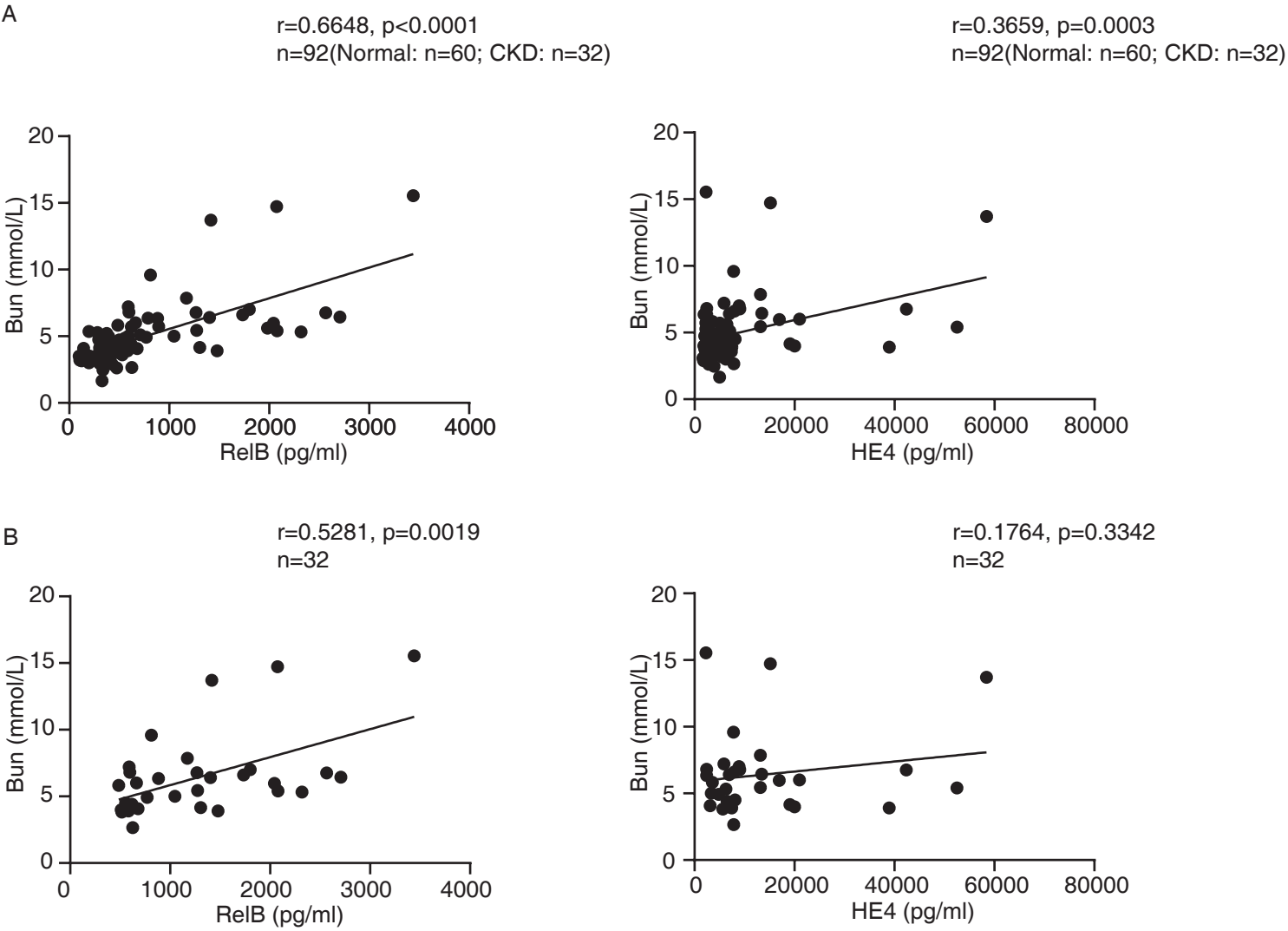

Supplemental Figure 4

A

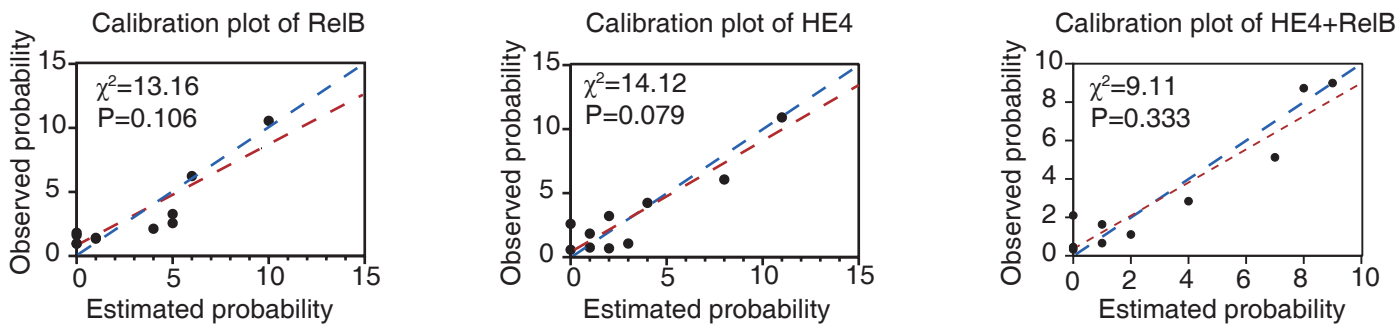

B

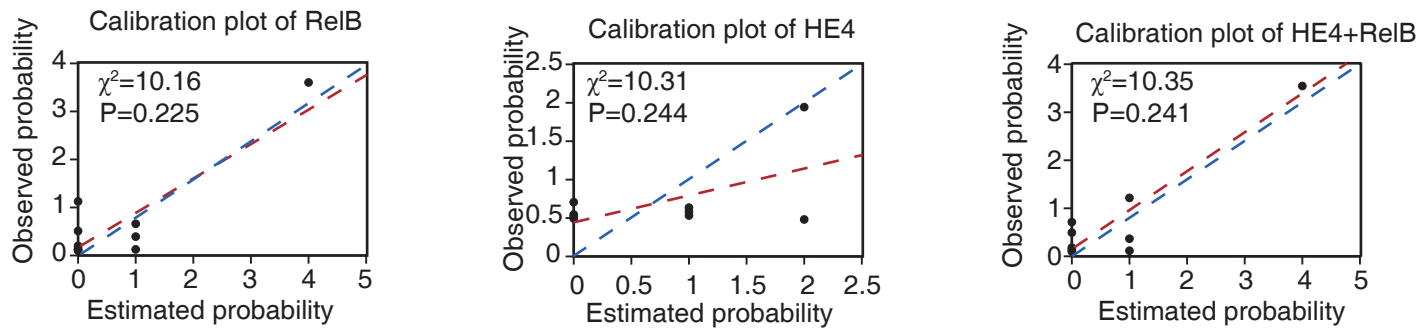

Supplement: Supplementary file 2 — Supplemental Figure 1. Altered expression of genes in NF‐κB and TGF‐β signaling pathways between UUO groups and sham group. The heatmap shows the renal tissue transcriptome‐wide changes in gene expression among three different groups (sham‐operated group, 2 days post‐UUO group and 8 days post‐UUO group). Normalized read counts values are recorded. 34 genes related to NF‐κB and TGF‐β signaling pathways were analyzed from a set of RNA‐seq data available at the NCBI GEO repository. GEO accession: GSE79443. Supplemental Figure 2. The correlation between RelB and kidney fibrosis at mRNA levels and protein levels. (A, B, C) Scatter plot with linear regression shows a correlation between mRNA expression of RelB and kidney fibrosis in obstructive nephropathy after UUO. (D) Quantification of the RelB Western blot results. (E) Correlation between RelB protein levels measured by Western blot and kidney fibrosis. r = 0.8854; p = 0.0001; n = 12. (F) Correlation between renal RelB staining and serum RelB, r = 0.8260; p < 0.0001; n = 18. Data were exhibited as means ± S.D. *p < 0.05 and **p < 0.001. Supplemental Figure 3. The correlation between the serum RelB and BUN. (A) Scatter plot with linear regression shows the serum RelB and HE4 levels are positively correlated with BUN in healthy controls and CKD patients. (B) Scatter plot with linear regression shows the serum RelB levels are positively correlated with BUN in CKD patients. However, correlation analysis revealed no significant associations between the serum HE4 and BUN. The Spearman correlation coefficient (r) and p value are shown. Supplemental Figure 4. Calibration of the model. (A) The calibration curve and Hosmer‐Lemeshow test for the predictive ability of RelB (χ 2 = 13.16, p = 0.106), HE4 (χ 2 = 14.12, p = 0.079) and the RelB + HE4 combination (χ 2 = 9.11, p = 0.333) in healthy group and CKD patients demonstrated good agreement with observation. (B) Similarly, the predictive ability of the RelB (χ 2 = 10.61, p = 0.2 [file CTM2-11-e362-s001.pdf]
