# Supplementary material for: Serum RelB is correlated with renal fibrosis and predicts chronic kidney disease progression
Source: Clin Transl Med. 2021 May 21;11(5):e362. doi: 10.1002/ctm2.362 (PMC8140188; doi:10.1002/ctm2.362)
Supplement: Supplementary file 3 — Table S1 [file CTM2-11-e362-s002.docx]

**Supplemental Table 1. Biomarker performance of renal fibrosis in CKD.**

| **Variables** | **AUC** | **SE** | **95% CI** | **Cutoff** | **Sensitivity (%)** | **Specificity**  **(%)** | **YI** | **Hosmer-Lemeshow test** |
| --- | --- | --- | --- | --- | --- | --- | --- | --- |
| HE4 | 0.849 | 0.047 | 0.757- 0.942 | 6259.73 | 71.9 | 91.7 | 0.636 | P=0.079 |
| RelB | 0.873 | 0.04 | 0.795- 0.952 | 503.23 | 90.6 | 80 | 0.706 | P=0.106 |
| HE4+ RelB | 0.92 | 0.031 | 0.86- 0.981 | -0.8865 | 87.5 | 90 | 0.775 | P=0.333 |

SE, standard error; 95% CI, 95% confidence interval; YI, Youden’s index.
